# Superior short-term memory in APOE ε2 carriers across the age range

**DOI:** 10.1016/j.bbr.2020.112918

**Published:** 2021-01-15

**Authors:** Nahid Zokaei, Alexander G Board, Ellie Slavkova, Clare E Mackay, Anna Christina Nobre, Masud Husain

**Affiliations:** a Oxford Centre for Human Brain Activity, Wellcome Centre for Integrative Neuroimaging, Department of Psychiatry, University of Oxford, Oxford, OX3 7JX, UK; b Department of Experimental Psychology, University of Oxford, Oxford, OX1 3UD, UK; c Department of Psychiatry, University of Oxford, Oxford, OX3 7JX, UK; d Nuffield Department of Clinical Neurosciences, University of Oxford, Oxford, OX3 9DU, UK; e Oxford NIHR Biomedical Research Centre, UK

**Keywords:** Short-term memory, Apolipoprotein, APOE e2, Alzheimer's disease

## Abstract

The Apolipoprotein-E (APOE) gene is now known to be associated with individual differences in cognitive health in ageing. However, while the *APOE* ε4 allele confers significantly increased risk of developing Alzheimer’s disease (AD), the *APOE* ε2 allele is hypothesized to be protective against the development of AD. This is in line with neuroimaging and pathological findings associated with ε2 *APOE* allele, which go in the opposite direction to those observed in AD-related pathology. However, the precise impact of this allele on cognition remains inconclusive, with some small-cohort studies raising the possibility of an advantageous memory performance in these individuals. Here, we tested short-term memory (STM) performance in a large cohort of individuals, 300 of which were *ε2/ε3* carriers. Their performance was compared to 554 *ε3/ε3* carriers. We included participants from a wide age range spanning young, middle-aged and elderly adults. All of them performed a STM task that has previously been shown to be sensitive to subtle changes in memory in various patient and at-risk cohorts. Individuals carrying the *APOE*-*ε2* allele exhibited a significant memory advantage, regardless of STM task difficulty and across all ages. The observed memory advantage was present across the age range, suggestive of a phenotypical effect of this allele on cognition, possibly independent of any effects of this genetic allele that occur later life in these individuals.

## Highlights

• The Apolipoprotein-E (*APOE*) ε*2* allele is known to be protective against Alzheimer’s disease.
• We tested the effect of this allele on cognitive performance, as measured by a sensitive short-term memory task.
• A large cohort of genotyped participants performed this task remotely.
• ε2 carriers demonstrated superior memory performance in young, middle-aged, and older participants.

## 1 Introduction

The apolipoprotein-E (*APOE*) gene has been linked to individual differences in risk and resilience to neurodegeneration in ageing. Three alleles make up the variants of the gene. The ε3 allele is the most common variant and considered to be the population norm. The ε4 allele is present in approximately 14% of the population and confers a major risk factor for developing Alzheimer’s disease (AD) [1]. Accordingly, the ε4 allele has been the focus of research in the past few years (e.g. [1], [2], [3], [4], [5], [6], [7]). In contrast, the rarer ε2 *APOE* allele has been hypothesized to be protective against AD pathology [8,9]. Investigations involving the ε2 allele remain rare, and thus there is scant evidence pertaining to its consequences on brain or cognitive functions related to AD.

Some studies have reported that the ε2 allele is associated with *decreased* AD-related effects on the brain, with carriers having lower hippocampal atrophy [10], or larger hippocampal volume [11,12] and increased entorhinal cortical thickness [13,14], compared to both ε3 and ε4 carriers. Additionally, some investigations have concluded that the ε2 allele might confer a protective effect against amyloid deposition and neurofibrillary tangle (NFT) formation [15,16]. Overall, *APOE* ε2 allele carriers have an increased lifespan [17].

A small number of behavioural studies have focused on cognitive correlates of carrying the APOE ε2 allele. The results, however, have been mixed and inconsistent. Some have reported a positive association between the ε2 allele and healthy cognitive functions in advanced years [[18], [19], [20], [21], [22]], for example an increased ability to retain information in short- and long-term memories [[22], [23], [24], [25]]. In one study, ε2 allele carriers were also found to have superior verbal memory with increased recall scores on intermediate and long-term recall tasks compared to non-carriers [18]. Similar effects have been reported in younger ε2 carriers. In fact some investigators have reported that ε2 carriers demonstrated superior performance in long-term and short-term memory tasks (as measured by n-back tasks) as well as tests of executive function and attention across a wide age range (23–67 years) [26].

Other studies challenge the presence of any benefits of the ε2 allele [27,28], and some even suggest a cognitive disadvantage [29]. For example, it has been reported that compared to non-carriers, individuals with the ε2 allele performed significantly worse on standard memory and executive function tasks [12,30]. Moreover, in a report by Lancaster and colleagues (2016), middle-aged ε2 carriers had performance disadvantages on various aspects of sustained attention, with slower response times in identifying a target compared to both ε3 and ε4 carriers [31]. In line with these findings, it has been found that even though ε2 carriers had reduced risk of clinical dementia, compared to ε3 carriers, they still possessed increased plaque neuropathology [32] which in turn may impact their cognitive abilities.

The scarcity and inconsistencies in the literature may stem from a number of shortcomings. They might be due to a lack of sensitivity in commonly used neuropsychological tests, variations in the age groups tested, as well as the small number of participants within each genetic group. At this point, it is crucial to resolve inconsistencies and to strive for a better description of the influences of the *APOE* ε2 gene allele on cognition. Understanding whether the ε2 is in fact protective of cognitive decline will help advance our knowledge about the genetic and neural factors promoting cognitive resilience and ultimately improve stratification, diagnosis, and prognosis of cognitive decline in neurodegeneration. In addition, there are practical consequences for understanding cognitive decline in ε4 carriers. Most studies investigating ε4 carriers as an at-risk group for developing AD compare them to ε2 and ε3 carriers as control participants. This might result in inconsistent findings if ε2 and ε3 carriers have distinct cognitive profiles.

To overcome these shortcomings, we employed a highly sensitive task of visual short-term memory (STM) to test a large cohort of APOE ε2 carriers and non-carriers online. Our task provides a specific and quantifiable measure of the quality of the memories formed and has been shown to be more sensitive than commonly used neuropsychological measures [33]. It has also been applied to APOE ε4 carriers, revealing an interesting pattern of antagonistic pleiotropy [2,3], thereby demonstrating the sensitivity of this task to detect subtle differences in performance in otherwise healthy participants. Secondly, to determine whether putative cognitive differences were part of individuals’ make-up or emerged gradually relative to non-carriers with advancing age, the large cohort included adults of various ages, spanning the young, middle-aged and elderly. Lastly, a large cohort of genotyped participants took part in online, with greater numbers of *APOE* ε2 carrier and non-carriers in each age-range than tested to date. The present study therefore allowed us to examine the effect of APOE ε2 allele on cognition, across the age range, and with high sensitivity.

## 2 Methods

Experimental procedures were reviewed and approved by the Central University Research Ethics Committee of the University of Oxford (identical to that detailed in 34).

### 2.1 Participants

Overall, 854 participants selected from a group of 1277 individuals recruited through the NIHR BioResources (https://bioresource.nihr.ac.uk/) completed the study remotely. Genetic information regarding the participants’ *APOE* allelic variants was used to select the group of participants, which consisted of 300 *ε2/ε3* carriers and 554 *ε3/ε3* carriers (for APOE genotyping methods please refer to the NIHR BioResources website: https://bioresource.nihr.ac.uk/). Participants were specifically recruited with the aim of testing as many individuals as possible within each genotype group and age from those available in the cohort. Hence, the distribution of participants in each genotype does not represent what one would expect in the general population.

On agreeing to take part, participants received a unique identification number and a guide describing the study procedures. They were first instructed to complete a consent form, followed by a set of demographic questionnaires. Having consented, participants then completed the Oxford Memory test (OMT) on their personal tablet devices.

Table 1 presents a summary of the demographic information for the two groups (age-related changes in memory performance and the influence of the *APOE ε4* was examined in a separate study 34). There was no significant difference in years of education (measured from the first year of school), gender, or handedness between the two groups.

**Table 1:** Demographic characteristics of *ε3/ε3 (black) and ε2/ε3* carriers (light blue).

### 2.2 Oxford memory test (OMT)

The Oxford Memory Test (OMT) is a flexible web-based version of a highly sensitive short-term-memory precision task we have previously successfully used to detect subtle changes in performance in healthy ageing, neurodegenerative disorders, and at-risk populations [2,[35], [36], [37]]. The online OMT instantiation is identical to that used in a previous study of APOE *ε4* carriers [34]. The web-app platform is designed for testing in ‘less strict’ environments such as clinics, wards, or individuals’ homes; and can be run on any tablet or touchscreen device.

**Fig. 1:**
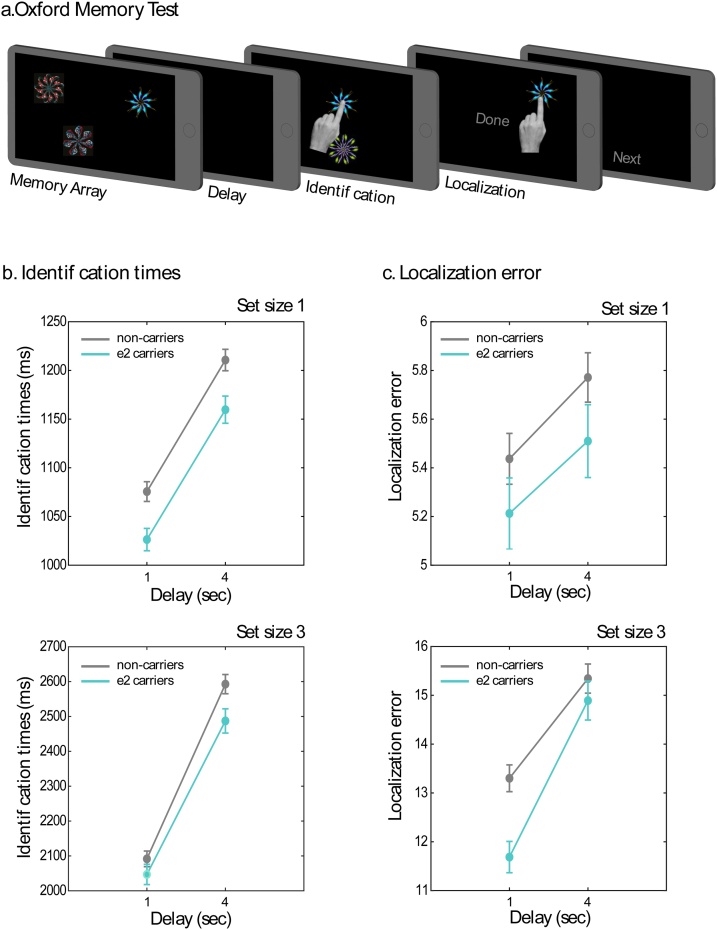
Short-term memory task and performance in *APOE ε2/ε3* and *ε3/ε3* carriers. a) Schematic of the short-term-memory task delivered via the OMT app. b) Identification times – the time it took participants to select the target item at response – for memory set sizes 1 and 3. *APOE ε2/ε3* carriers were significantly faster than non-carriers. c) Localization error – the distance between the reported location of the target and the true location of the item at memory array – for memory set sizes 1 and 3. Similarly, *APOE ε2/ε3* carriers were significantly more precise than non-carriers.

A schematic of the task is presented in Fig. 1a. In each trial, participants viewed 1 or 3 coloured fractals (memory array) for 1 or 3 s respectively and had to memorise the identity and corresponding location of each fractal. The memory array was followed by a blank delay of 1 or 4 s before they were probed about their memory. At probe, participants were first presented with 2 fractals, positioned above and below the fixation cross. One was from the memory array (target) and the other was a novel fractal. Participants had to select the fractal that had been present in the previous memory array (identification) and then drag it to the remembered location (localization). To confirm their response, participants then had to press the “Done” button at screen centre. This was followed by the presentation of a blank screen with a “Next” button (Fig. 1a). When ready, participants initiated the next trial.

The stimuli were selected from a pool of 196 coloured fractals (sized to 3° of visual angle). The location of the fractals in the memory array was randomly selected with a few constraints: Fractals had a minimum distance of 4° of visual angle from each other, a minimum of 1.5° of visual angle from the edges of the screen, and a minimum of 2° of visual angle from screen centre, assuming a constant viewing distance of 40 cm.

Participants were asked to complete 2 blocks of 40 trials. Each block consisted of 10 trials per memory set-size and delay condition. Prior to the beginning of the task, participants were acquainted with the experimental design and conditions. They completed 2 trials with written instructions on each screen and a further 8 practice trials resembling the experimental trials. Practice trials were not included in the analysis. Participants were instructed to perform the task in a quiet place while placing the tablet at arm’s length. Prior to the start of the task, participants were asked to report the screen dimensions within the OMT app. This information was used to normalise screen sizes across various devices.

## 3 Results

Mixed ANOVAs with the number of objects in the memory array (set size) and the duration of the delay period as within-subject factors and age-group and *APOE* gene-status as between-subject factors were conducted (see Table 2 for complete summary statistics). In summary, the analysis showed significant performance benefits for *APOE* ε2 carriers, discussed below, none of which interacted with age of participants.

**Table 2:** Summary statistics on performance in the STM task for carriers and non-carriers of the *APOE ε2/ε3* gene allele in different age groups (significant values are highlighted in bold).

For *identification times* (time participants took to select the target fractal at response), there was a significant main effect of *APOE* status (*F*(1,840) = 4.75, *p* = 0.03, η^2^_p_ = 0.006, Fig. 1b), with faster responses by APOE *ε2/ε3* carriers than non-carriers. *APOE* status, however, did not interact with any of the other factors, i.e. age, delay interval or set size (Table 2).

For *Identification accuracy*, there was no significant main effect of *APOE* status or an interaction between *APOE* status or any of the other factors. Mean identification accuracy was high overall (mean accuracy of 99% for set size 1 and 88% for set size 3), and interacted with age of participants as well as memory delay and set size (see Table 2 for summary statistics).

Finally, there was a significant main effect of *APOE* status for *localization error* – the distance between the response location and the original location of the probed item (*F*(1,840) = 5.9, *p* < 0.001, η^2^_p_ = 0.007), with *APOE ε2/ε3* carriers localizing fractals more precisely than non-carriers. *APOE* status also interacted significantly with set size (*F*(1,840) = 4.3, *p* = 0.038, η^2^_p_ = 0.005). This 2-way interaction was followed up by further one-way analyses per set size. For set size 1, there was no significant effect of *APOE* status (*F*(1,840) = 1.85, *p* = 0.17) but for set size 3, there was a significant advantage for *ε2* carriers (*F*(1,840) = 6.02, *p* = 0.014, η^2^_p_ = 0.007- Fig. 1c). *APOE* status did not interact with age of participants or memory delay (Table 2).

Together, these results demonstrate that individuals with the *ε2/ε3* genotype performed significantly better in our STM task, as measured by both faster response times and greater precision of location memory compared to the *ε3/ε3* genotype, regardless of age.

## 4 Discussion

The current study provides evidence for a distinct pattern of STM performance in *ε2* carriers compared to non-carriers. Specifically, *ε2* carriers were faster at identifying the target item and then placed the chosen item more accurately at the remembered location. Importantly, this memory advantage was observed regardless of age of participants, with significant cognitive differences detectable even in young adults. These findings provide evidence for the influences of the *APOE* ε2 gene allele on cognition, which in turn can inform studies investigating cognitive biomarkers for AD in ε4 carriers. Traditionally, many studies have grouped the ε2/ε3 and ε3/ε3 genotypes into one “control” group to compare to the at-risk ε4-carrier cohort [7,[38], [39], [40]]. However, as demonstrated here, due to the distinct nature of their cognitive profiles, collapsing these two groups could introduce unplanned variability. Differential inclusion of ε2 carries may therefore have contributed to the inconsistencies reported in the *APOE* ε4 literature (e.g. 3,[37], [38], [39], [40], [41], [42]).

Investigations on the effects of the *ε2* allele on cognition have provided mixed results [[18], [19], [20], [21], [22], [23], [24], [25], [26], [27], [28], [29],[43], [44], [45]]. Our results complement and strengthen previous observations of superior memory performance in *ε2* carriers in studies with far smaller samples [25]. For example, in an investigation of both immediate and delayed verbal memory, middle-aged and older *ε2* carriers had better memory performance compared to non-carriers, who also experienced sharper decline in memory decay [18]. This effect remained significant even after controlling for the occurrence of cardiovascular disorders in all groups. Similarly, younger *ε2* carriers have been shown to have advantageous effects in tasks of both short- and long-term memories previously [26]. However, it is important to note that the effect sizes in the present study are small. Therefore, even though there is a consistent influence of the APOE *ε2* on performance, future attempts should identify the underlying biological mechanisms of these changes in cognition.

There are a number of possible explanations for better cognitive performance in *ε2* carriers, though they remain speculative. First, there is evidence that *ε2* carriers are more resistant to neurodegeneration, have more efficient clearance of amyloid from blood vessels [9,46], and are protected against neurofibrillary tangle formation [15,16]. Additionally, some studies have reported that *APOE ε2* carriers have lower levels of hippocampal atrophy in old age [10]. However, such mechanisms may not fully explain the pattern of results in the current study, since the cognitive benefits of the *APOE ε2* carriers were not restricted to older participants but also appeared in the younger participants. Our results suggest instead an alternative possibility, namely a phenotypic difference that is independent of side-effects of potential progression of pathology associated with age. Consistent with this view, larger hippocampal volumes have been reported in *APOE ε2* carriers even in younger participants [11,12]. It will be interesting in future studies to investigate the possible link between the observed behavioural advantage and hippocampal size and functional integrity.

Our results contrast with a number of previous studies reporting no advantageous memory performance in *ε2* carriers [12,27,28,30,31]. Many important factors may contribute to this discrepancy. To avoid the lack of power to detect genetic differences in small cohorts [25], we studied, to the best of our knowledge, one of the largest cohorts of *ε2* carriers. Our task also brought a significantly more sensitive task of STM compared to traditional and commonly used neuropsychological measures of memory used in many of the previous studies [33]. Finally, previous investigations have each targeted different age groups, limiting the ability to generalise any findings across the age range. In this study, by using online testing, we were able to test participants across a wide age span (20 s to 70 s).

Previously we have shown that *ε4* carriers can also demonstrate superior STM performance compared to *ε3/ε3* carriers across the age range, using an identical task [2,3,34]. Crucially, this effect was, however, only observed for the shorter memory delays of 1 s, with higher forgetting rate compared to non-carriers in STM as well as worse LTM performance in the same individuals [2]. The advantage in very short-term memories in *ε4* carriers was interpreted to reflect antagonistic pleiotropy effects of the APOE gene [47]. The overall beneficial memory performance in *ε2* carriers, however, may be a phenotypical effect of the *APOE* ε2 allele on cognition arising as a secondary consequence of other biological changes associated with this allele, e.g. associated with vascular regulation [9]. Future research might profitably focus on understanding the link between physiological and cognitive changes associated with the *APOE ε2* carriers. Specifically, a wider range of cognitive processes, beyond those examined here, should be tested to provide a comprehensive cognitive landscape of *APOE ε2* carriers, the relationship between processes and their link to physiological changes.

Together, the findings presented here provide evidence for beneficial effects of the *APOE ε2* gene allele on memory in otherwise healthy participants, across ageing. To the best of our knowledge, this is one of the first studies to test memory in a large sample of participants, using a sensitive task, and including individuals across a large age span, overcoming possible shortcomings of previous investigations into the topic. Future research should aim to replicate these findings in the general population, limiting any possible selection biases that may have influenced the findings. Further, it would be important to identify the biological basis of such changes in cognition as a result of different variants of the *APOE* gene. Such considerations will be crucial in developing a thorough understanding of the protective vs. detrimental nature of different APOE alleles on neurodegeneration and brain health in general.

## CRediT authorship contribution statement

**Nahid Zokaei:** Conceptualization, Data curation, Formal analysis, Funding acquisition, Investigation, Methodology, Project administration, Resources, Software, Supervision, Validation, Visualization, Writing - original draft. **Alexander G Board:** Data curation, Formal analysis, Writing - review & editing. **Ellie Slavkova:** Project administration, Data curation. **Clare E Mackay:** Supervision, Writing - review & editing. **Anna Christina Nobre:** Conceptualization, Funding acquisition, Supervision, Writing - review & editing. **Masud Husain:** Conceptualization, Funding acquisition, Supervision, Writing - review & editing.

## References

[1] Liu C.-C., Liu C.-C., Kanekiyo T., Xu H., Bu G. (2013). Apolipoprotein E and Alzheimer disease: risk, mechanisms and therapy. Nat. Rev. Neurol..

[2] Zokaei N., Čepukaitytė G., Board A.G., Mackay C.E., Husain M., Nobre A.C. (2019). Dissociable effects of the apolipoprotein-E (APOE) gene on short- and long-term memories. Neurobiol. Aging.

[3] Zokaei N., Giehl K., Sillence A., Neville M.J., Karpe F., Nobre A.C. (2017). Sex and APOE: a memory advantage in male APOE ε4 carriers in midlife. Cortex J. Devoted Study Nerv. Syst. Behav..

[4] Heise V., Filippini N., Trachtenberg A.J., Suri S., Ebmeier K.P., Mackay C.E. (2014). Apolipoprotein E genotype, gender and age modulate connectivity of the hippocampus in healthy adults. NeuroImage.

[5] Farrer L.A., Cupples L.A., Haines J.L., Hyman B., Kukull W.A., Mayeux R. (1997). Effects of age, sex, and ethnicity on the association between apolipoprotein E genotype and Alzheimer disease. A meta-analysis. APOE Alzheimer Dis. Meta Anal. Consortium. JAMA.

[6] Filippini N., Ebmeier K.P., MacIntosh B.J., Trachtenberg A.J., Frisoni G.B., Wilcock G.K. (2011). Differential effects of the APOE genotype on brain function across the lifespan. NeuroImage.

[7] Agosta F., Vossel K.A., Miller B.L., Migliaccio R., Bonasera S.J., Filippi M. (2009). Apolipoprotein E epsilon4 is associated with disease-specific effects on brain atrophy in Alzheimer’s disease and frontotemporal dementia. Proc. Natl. Acad. Sci. U. S. A..

[8] Lee G., Pollard H.B., Arispe N. (2002). Annexin 5 and apolipoprotein E2 protect against Alzheimer’s amyloid-β-peptide cytotoxicity by competitive inhibition at a common phosphatidylserine interaction site. Peptides.

[9] Suri S., Heise V., Trachtenberg A.J., Mackay C.E. (2013). The forgotten APOE allele: a review of the evidence and suggested mechanisms for the protective effect of APOE ε2. Neurosci. Biobehav. Rev..

[10] Chiang G.C., Insel P.S., Tosun D., Schuff N., Truran-Sacrey D., Raptentsetsang S.T. (2010). Hippocampal atrophy rates and CSF biomarkers in elderly APOE2 normal subjects. Neurology.

[11] Konishi K., Bhat V., Banner H., Poirier J., Joober R., Bohbot V.D. (2016). APOE2 is associated with spatial navigational strategies and increased gray matter in the Hippocampus. Front. Hum. Neurosci..

[12] Alexopoulos P., Richter-Schmidinger T., Horn M., Maus S., Reichel M., Sidiropoulos C. (2011). Hippocampal volume differences between healthy young apolipoprotein E ε2 and ε4 carriers. J Alzheimers Dis. JAD..

[13] Shaw P., Lerch J.P., Pruessner J.C., Taylor K.N., Rose A.B., Greenstein D. (2007). Cortical morphology in children and adolescents with different apolipoprotein E gene polymorphisms: an observational study. Lancet. Neurol..

[14] Bunce D., Anstey K.J., Cherbuin N., Gautam P., Sachdev P., Easteal S. (2012). APOE genotype and entorhinal cortex volume in non-demented community-dwelling adults in midlife and early old age. J. Alzheimers Dis. JAD..

[15] Nagy Z., Esiri M.M., Jobst K.A., Johnston C., Litchfield S., Sim E. (1995). Influence of the apolipoprotein E genotype on amyloid deposition and neurofibrillary tangle formation in Alzheimer’s disease. Neuroscience.

[16] Oyama F., Shimada H., Oyama R., Ihara Y. (1995). Apolipoprotein E genotype, Alzheimer’s pathologies and related gene expression in the aged population. Brain Res. Mol. Brain Res..

[17] Blanché H., Cabanne L., Sahbatou M., Thomas G. (2001). A study of French centenarians: are ACE and APOE associated with longevity?. C. R. Acad. Sci. III.

[18] Helkala E.L., Koivisto K., Hänninen T., Vanhanen M., Kervinen K., Kuusisto J. (1995). The association of apolipoprotein E polymorphism with memory: a population based study. Neurosci. Lett..

[19] Helkala E.L., Koivisto K., Hanninen T., Vanhanen M., Kervinen K., Kuusisto J. (1996). Memory functions in human subjects with different apolipoprotein E phenotypes during a 3-year population-based follow-up study. Neurosci. Lett..

[20] Hyman B.T., Gomez-Isla T., Rebeck G.W., Briggs M., Chung H., West H.L. (1996). Epidemiological, clinical, and neuropathological study of apolipoprotein E genotype in Alzheimer’s disease. Ann. N. Y. Acad. Sci..

[21] Staehelin H.B., Perrig-Chiello P., Mitrache C., Miserez A.R., Perrig W.J. (1999). Apolipoprotein E genotypes and cognitive functions in healthy elderly persons. Acta Neurol. Scand..

[22] Wilson R.S., Bienias J.L., Berry-Kravis E., Evans D.A., Bennett D.A. (2002). The apolipoprotein E epsilon 2 allele and decline in episodic memory. J. Neurol. Neurosurg. Psychiatry.

[23] Mondadori C.R.A., de Quervain D.J.-F., Buchmann A., Mustovic H., Wollmer M.A., Schmidt C.F. (2007). Better memory and neural efficiency in young apolipoprotein E epsilon4 carriers. Cereb Cortex N Y N 1991.

[24] Small B.J., Rosnick C.B., Fratiglioni L., Bäckman L. (2004). Apolipoprotein E and cognitive performance: a meta-analysis. Psychol. Aging.

[25] Wisdom N.M., Callahan J.L., Hawkins K.A. (2011). The effects of apolipoprotein E on non-impaired cognitive functioning: a meta-analysis. Neurobiol. Aging.

[26] Sinclair L.I., Pleydell-Pearce C.W., Day I.N.M. (2017). Possible positive effect of the APOE ε2 allele on cognition in early to mid-adult life. Neurobiol. Learn. Mem..

[27] Marioni R.E., Campbell A., Scotland G., Hayward C., Porteous D.J., Deary I.J. (2016). Differential effects of the APOE e4 allele on different domains of cognitive ability across the life-course. Eur. J. Hum. Genet. EJHG..

[28] Meyer M.R., Tschanz J.T., Norton M.C., Welsh-Bohmer K.A., Steffens D.C., Wyse B.W. (1998). APOE genotype predicts when--not whether--one is predisposed to develop Alzheimer disease. Nat. Genet..

[29] Lancaster C., Forster S., Tabet N., Rusted J. (2017). Putting attention in the spotlight: the influence of APOE genotype on visual search in mid adulthood. Behav. Brain Res..

[30] Alexander D.M., Williams L.M., Gatt J.M., Dobson-Stone C., Kuan S.A., Todd E.G. (2007). The contribution of apolipoprotein E alleles on cognitive performance and dynamic neural activity over six decades. Biol. Psychol..

[31] Lancaster C., Tabet N., Rusted J. (2016). The APOE paradox: do attentional control differences in mid-adulthood reflect risk of late-life cognitive decline. Neurobiol. Aging.

[32] Berlau D.J., Corrada M.M., Head E., Kawas C.H. (2009). APOE epsilon2 is associated with intact cognition but increased Alzheimer pathology in the oldest old. Neurology.

[33] Zokaei N., Burnett Heyes S., Gorgoraptis N., Budhdeo S., Husain M. (2014). Working memory recall precision is a more sensitive index than span. J. Neuropsychol..

[34] Zokaei N., Grogan J., Fallon S.J., Slavkova E., Hadida J., Manohar S. (2020). Short-term memory advantage for brief durations in human APOE ε4 carriers. Sci. Rep..

[35] Pertzov Y., Miller T.D., Gorgoraptis N., Caine D., Schott J.M., Butler C. (2013). Binding deficits in memory following medial temporal lobe damage in patients with voltage-gated potassium channel complex antibody-associated limbic encephalitis. Brain. J. Neurol..

[36] Liang Y., Pertzov Y., Nicholas J.M., Henley S.M.D., Crutch S., Woodward F. (2016). Visual short-term memory binding deficit in familial Alzheimer’s disease. Cortex J. Devoted Study Nerv. Syst. Behav..

[37] Zokaei N., Nour M.M., Sillence A., Drew D., Adcock J., Stacey R. (2018). Binding deficits in visual short-term memory in patients with temporal lobe lobectomy. Hippocampus.

[38] Wolk D.A., Dickerson B.C. (2010). Alzheimer’s disease neuroimaging Initiative. Apolipoprotein E (APOE) genotype has dissociable effects on memory and attentional-executive network function in Alzheimer’s disease. Proc. Natl. Acad. Sci. U. S. A.

[39] Greenwood P.M., Espeseth T., Lin M.-K., Reinvang I., Parasuraman R. (2014). Longitudinal change in working memory as a function of APOE genotype in midlife and old age. Scand. J. Psychol..

[40] Greenwood P.M., Lambert C., Sunderland T., Parasuraman R. (2005). Effects of apolipoprotein E genotype on spatial attention, working memory, and their interaction in healthy, middle-aged adults: results from the National Institute of Mental Health’s BIOCARD study. Neuropsychology.

[41] Evans S., Dowell N.G., Tabet N., Tofts P.S., King S.L., Rusted J.M. (2014). Cognitive and neural signatures of the APOE E4 allele in mid-aged adults. Neurobiol. Aging.

[42] Flory J.D., Manuck S.B., Ferrell R.E., Ryan C.M., Muldoon M.F. (2000). Memory performance and the apolipoprotein E polymorphism in a community sample of middle-aged adults. Am. J. Med. Genet..

[43] Levy J.A., Bergeson J., Putnam K., Rosen V., Cohen R., Lalonde F. (2004). Context-specific memory and apolipoprotein E (ApoE) epsilon 4: cognitive evidence from the NIMH prospective study of risk for Alzheimer’s disease. J. Int. Neuropsychol. Soc JINS.

[44] Nilsson L.-G., Adolfsson R., Bäckman L., Cruts M., Nyberg L., Small B.J. (2006). The influence of APOE status on episodic and semantic memory: data from a population-based study. Neuropsychology.

[45] Sager M.A., Hermann B., La Rue A. (2005). Middle-aged children of persons with Alzheimer’s disease: APOE genotypes and cognitive function in the Wisconsin Registry for Alzheimer’s Prevention. J. Geriatr. Psychiatry Neurol..

[46] Conejero-Goldberg C., Gomar J.J., Bobes-Bascaran T., Hyde T.M., Kleinman J.E., Herman M.M. (2014). APOE2 enhances neuroprotection against Alzheimer’s disease through multiple molecular mechanisms. Mol. Psychiatry.

[47] Williams G.C. (1957). Pleiotropy, natural selection, and the evolution of senescence. Evolution.

